# Multifaceted Applications of Synthesized Silver Nanoparticles From Marine Fungus *Aspergillus flavus* MK4: Antimicrobial, Anticancer, and Wound‐Healing Properties

**DOI:** 10.1155/bmri/7161915

**Published:** 2025-08-27

**Authors:** Mohamed H. Al-Agamy, Mahmoud S. Kelany, Sultan Alshehri, Mohammad R. Alhuzani, Moaz M. Hamed, Wael Sabra, Wael A. Mahdi

**Affiliations:** ^1^ Department of Pharmaceutics, College of Pharmacy, King Saud University, Riyadh, Saudi Arabia, ksu.edu.sa; ^2^ Microbiology Department, Marine Environment Division, National Institute of Oceanography and Fisheries (NIOF), Cairo, Egypt, azhar.edu.eg; ^3^ Faculty of Life Sciences, Rhine-Waal University of Applied Sciences, Kleve, Germany, hochschule-rhein-waal.de

**Keywords:** antimicrobial activity, antioxidant activity, biosynthesis, cytotoxic activity, marine fungi, silver nanoparticle

## Abstract

Due to their wide range of applications in modern technology, silver metallic nanomaterials have garnered considerable attention, driving significant research and development efforts. This study focuses on investigating the antimicrobial properties of silver nanoparticles (Ag‐NPs), encompassing their production, characterization, and biological aspects. The eco‐friendly extracellular biosynthetic method employed in this work utilizes extracts from the marine fungus *Aspergillus flavus* MK4 as reducing agents for nanoparticle synthesis. The analysis of colloidal Ag‐NPs using ultraviolet‐visible spectroscopy revealed a peak in absorbance at 460 nm, indicating the presence of plasmonic properties. Electron imaging of the internal structure (TEM) elucidated the spherical shape of Ag‐NPs, which measure 15 nm in size. Testing against bacterial diversities revealed potent antibacterial and antifungal efficacy. Cytotoxicity against the HepG‐2 cell line was assessed using the MTT assay, and antioxidant properties were examined through the radical scavenging (DPPH) assay. Based on their conceivable applications in the fields of antibacterial, anticancer, and wound healing, the synthesized Ag‐NPs exhibit promising features. With its powerful ability to synthesize Ag‐NPs, *A. flavus* MK4 can generate extensively characterized nanoparticles, demonstrating their antibacterial, antioxidant, anticancer, and wound‐healing properties.

## 1. Introduction

The term “marine fungi” encompasses a diverse range of fungi that typically inhabit natural environments in marine and estuarine settings [1]. Fungi that inhabit marine environments can be found in a wide variety of habitats within each marine ecosystem. The fact that they establish crucial symbiotic connections with other species, such as algae and sponges, gives them significance that extends beyond their mere presence. Marine fungi play a vital role in recycling nutrients and carbon‐based compounds, contributing to the maintenance of healthy marine ecosystems and ensuring their proper functioning [2].

Ascomycota, Basidiomycota, and Chytridiomycota are the three basic phyla that are considered to be included in the realm of marine fungi currently being studied by science. These fungi can be found in a wide range of marine ecosystems, spanning the open ocean to sediments, mangroves, and coral reefs. Numerous marine fungi have developed special adaptations that allow them to flourish in environments that are submerged in water. The osmoregulatory systems that these fungi have developed allow them to maintain the stability of their internal salt concentrations despite the fact that the surrounding conditions are constantly changing. In addition to this, they are capable of producing secondary metabolites, such as polyketides and terpenoids, which possess antibacterial, antiviral, and antifungal effects [3].

There is no doubt that nanotechnology stands out as one of the technologies that is extremely promising and is progressing at a rapid pace. In recent decades, nanoparticles have garnered significant interest due to their distinct properties in areas such as optics, chemistry, magnetism, biology, and electricity. These properties differ from those observed in their corresponding bulk materials [4]. Their high surface‐area‐to‐volume ratio, stemming from their compact structure, facilitates significant interactions and enhances responsiveness. In addition, the fact that they are so small makes it simple to modify their surface qualities, which enables them to be tailored to specific applications using the properties. All of these characteristics, taken together, lead to the extraordinary adaptability of nanoparticles and the potential uses they have in a wide range of scientific, industrial, and medical domains [5]. Silver nanoparticles (Ag‐NPs) have attracted significant attention because of their distinctive physicochemical characteristics and diverse applications in fields such as medicine, electronics, and environmental remediation. The appeal of marine fungi lies in their potential to serve as a bioactive chemical source, making them especially attractive for synthesis of Ag‐NPs. The combination of marine fungi and Ag‐NPs presents a substantial opportunity to improve the production of these nanoparticles, which can lead to unique features and a wide range of applications in various fields [6]. A wide range of industries, including agriculture, commerce, medicine, and manufacturing, are already making extensive use of Ag‐NPs through their ubiquitous application. Due to their diverse qualities, they can be applied across a wide range of sectors and in various applications [7].

Ag‐NPs have diverse applications in medicine, serving as enhancers for vaccines, drugs for diabetes, agents for wound and bone healing, components in biosensors, and contributors to anticancer treatment. The unique features of Ag‐NPs emphasize their importance in medical applications, highlighting the prospective role they play in advancing healthcare and medical interventions [8]. Marine fungi facilitate the biosynthetic process that transforms silver ions (Ag^+^) into elemental silver (Ag^0^) through the generation of Ag‐NPs. The enzymes and/or metabolites produced by the fungi play a crucial role in aiding this conversion. Although the entire mechanism of Ag‐NP biosynthesis remains incompletely understood, it is thought to entail the reduction of Ag^+^ to Ag^0^ [8–10].

Several marine fungi, including *Aspergillus*, *Penicillium*, *Fusarium*, and *Trichoderma*, have been identified as biosynthesized Ag‐NP. Researchers have employed various characterization methods to analyze these biosynthesized Ag‐NPs. Several methods, such as UV‐vis spectroscopy, transmission electron microscopy (TEM), x‐ray diffraction (XRD), and Fourier transform infrared spectroscopy (FTIR), are employed to gain valuable information on the physical, chemical, and structural characteristics of Ag‐NP generated by marine fungi [9]. Moreover, Ag‐NPs produced by the biosynthesis process by marine fungi have exhibited potent antibacterial, antioxidant, and anticancer properties. This makes them highly attractive for many medical applications [11].

Our aim in this research is to synthesize Ag‐NPs utilizing cell‐free filtrate (CFF) derived from the marine fungus *Aspergillus flavus*. The morphological and structural features of the generated nanoparticles were extensively analyzed using methods such as UV‐vis spectroscopy, TEM imaging, FTIR, and XRD. The antibacterial effectiveness of Ag‐NPs was investigated after synthesis by exposing them to different pathogenic bacteria. The antioxidant properties of Ag‐NPs were assessed by the 2,2‐diphenyl‐1‐picrylhydrazyl (DPPH) assay. Additionally, the cytotoxicity of Ag‐NPs was examined.

## 2. Materials and Methods

### 2.1. Tested Bacterial Isolates Which Were Used in Antimicrobial Test

*Bacillus subtilis* ATCC 6633, *Staphylococcus aureus* ATCC 25923, and *Bacillus cereus* are examples of gram‐positive strains. Additionally, there are gram‐negative strains, including *Pseudomonas aeruginosa* ATCC 9027, *Salmonella typhimurium* ATCC 14028, *Pseudomonas fluorescens* DSM 50090, and *Aeromonas hydrophila* NRRL 914.

### 2.2. Tested Fungal Isolates Used in Antifungal Tests

Fungal strains NIOF‐F3, NIOF‐F8, NIOF‐F12, NIOF‐F13, NIOF‐F15, NIOF‐F22, NIOF‐F48, NIOF‐F63, and NIOF‐F71, namely, *Aspergillus fumigatus*, *Aspergillus niger*, *A. flavus*, *Aspergillus terreus*, *Aspergillus parasiticus*, *Penicillium oxalicum*, *Fusarium solani*, *Fusarium oxysporum*, and *Candida albicans* [12], were sourced from the NIOF Microbiological Lab at the NIOF Red Sea branch in Egypt. These marine pathogenic strains were maintained on YPD agar slants (yeast extract peptone dextrose) and preserved for an extended period at approximately 4°C by folding the slants with 25% glycerol.

### 2.3. Sampling and Fungal Isolation

Aseptic procedures were used to collect sediment samples from various locations, including the Suez Gulf, the city of Hurghada, the National Institute and the Beach of Oceanography. The specific coordinates for these locations are 27° 17 ^′^ 03 ^″^ N latitude and 33° 46 ^′^ 15 ^″^ E longitude. The sampling focused on the plow layer, spanning depths from 0 to 25 cm. Each sediment sample, weighing approximately 100 g, was meticulously deposited in sterile vials, each appropriately labeled [13]. The isolation of fungi involved the use of the serial dilution technique. Initially, a gram of sediment sample was suspended in 10 mL of sterile seawater, resulting in the generation of serial dilutions ranging from 10^−1^ to 10^−5^. The next step was to inoculate modified potato dextrose agar (MPDA) medium (containing potatoes [sliced washed unpeeled], 4 g [from 200 g of infused potato]; dextrose, 20 g; dipotassium hydrogen phosphate, 1.0; potassium nitrate, 2.0; magnesium sulfate, 0.5; agar, 18.0; and distilled water, 1 L with 1% streptomycin) with 1 mL of each dilution. After inoculation, the medium petri dishes were transferred to an incubator set at a temp. of 28 ± 2^°^C, where they were allowed to remain undisturbed for a duration of 14 days. Throughout the weeklong observation period, fungal colonies were carefully monitored. Pure cultures of observed fungal colonies were identified and maintained for further analysis [14].

### 2.4. Preparation of Cell‐Free Extract

To create the inoculum for the fungal broth, a loop carrying approximately 1 × 10^6^ spores of selected isolates was transferred to 50 mL of modified potato starch (MPD) broth medium. The transfer was executed using a 250‐mL Erlenmeyer flask. To synthesize Ag‐NPs, a fungal inoculum was introduced into a 250‐mL conical flask filled with MPD broth. The flask was incubated on a rotary shaker at 220 rpm for 5 days at a temperature of 28 ± 2^°^C. Subsequently, the culture was subjected to centrifugation at 10,000 rpm to collect the supernatant, a critical component necessary for the synthesis of Ag‐NPs [15].

### 2.5. Green Synthesis of Ag‐NPs

The supernatants, also known as biomass filtrate, were engaged in the synthesis of environmentally friendly Ag‐NPs. In 100‐mL Erlenmeyer flasks, 30 mL of 10 mmole AgNO_3_ were combined with 90 mL of isolated supernatant. Incubation of the flasks was carried out at 28 ± 2^°^C, and observations were made for any color changes. Two controls were implemented for comparison. In the initial control, the sterile media was mixed with 10 mM silver nitrate; this control served to validate that the media components alone were incapable of the transformation of Ag^+^ to nanomaterials containing silver. In the negative control, which consisted of a silver nitrate solution, no observable color change occurred over time. Daily monitoring of the flasks was carried out to detect any visual alterations in color. Additionally, UV‐visible spectroscopy, covering the range of 200–800 nm, was performed for flasks that showed noticeable color adjustments. This analytical approach provides a detailed examination of the spectral characteristics associated with the synthesis of Ag‐NPs [16].

### 2.6. Characterizations of Ag‐NPs

We used a METASHUV‐9000A UV‐visible double‐beam spectrophotometer to analyze the absorption spectra of the generated (Ag‐NPs) over the wavelength range from 200 to 800 nm. To assess the morphology and dimensions of the synthesized Ag‐NPs, TEM and scanning electron microscopy (SEM) were employed. The SEM analysis was performed to determine the size, shape, and morphology of the manufactured nanoparticles. A specimen was prepared by placing a drop of the solution onto a copper grid coated with carbon. Following this, any surplus solution was discarded, and the sample was left to cure naturally. The SEM research was observed at the Regional Center for Mycology and Biotechnology at Al‐Azhar University, Egypt, using a JEOL JSM‐5500LV Scanning Electron Microscope (JEOL Instruments Inc., Japan) at an accelerating voltage of 20 kV [17]. Samples for TEM characterization were prepared by placing a drop of the Ag‐NP suspension on a carbon‐coated copper grid and dried at room temperature. Samples were examined with a JEOL 1010 Transmission Electron Microscope (JEOL Ltd., Tokyo, Japan) at 80 kV, and digital photographs were taken using a Hamamatsu digital camera C4742‐57‐12NR (Hamamatsu, Japan) at the same scientific center. Moreover, an x‐ray diffractometer was utilized to generate an XRD pattern of Ag‐NPs. The Shimadzu XRD‐7000 instrument was employed to produce an XRD pattern of Ag‐NPs, with the analysis conducted in transmission mode. The measurements were carried out at the Central Laboratory of the City of Scientific Research and Technological Applications in Egypt. CuK*α* radiation with a wavelength of 1.5406 nm was utilized, and the measurements were performed within the 2*θ* range of 0° to 90°, operating at 30 kV and 100 mA [10].

### 2.7. Genotypic Characterization of a Potent Isolate

The genomic DNA extraction from isolate (MK4) used the cellular DNeasy Kit, following the standard instructions. For the fungi, genomic DNA extraction was performed from cultures with 7 days of age using the DNeasy technologies provided by QIAGEN. Amplification of ribosomal spacer DNA (ITS) was carried out using primers ITS1 and ITS4. Subsequently, the purification of the PCR products was accomplished using the QIAquick Kit [18]. The resulting PCR products were sent to Color Lab., Cairo, for sequencing. The sequences obtained underwent a BLAST search in GenBank (NCBI) for comparison with other relevant sequences.

### 2.8. Antimicrobial Activity of Ag‐NPs

The well diffusion method was used to assess the antibacterial activity of the synthesized Ag‐NPs [19]. For all test bacterial strains, an overnight growth at 37°C in nutrient broth was used with a McFarland 0.5 adjustment. Subsequently, 100 *μ*L from the tested isolates was spread aseptically on individual nutrient agar plates. Using a cork borer, an 8‐mm diameter well was created on the agar plate, and 60 *μ*L of the synthesized Ag‐NP was introduced into each well under sterile conditions. Furthermore, a positive control was established with 100 *μ*L of ampicillin (1 mg/mL), while negative controls comprised 60 *μ*L each of 1 mM silver nitrate and culture broth (culture without AgNO_3_). Subsequently, the plates were incubated for 24 h at 37°C, and the evaluation of antibacterial activity involved measuring the diameter of the inhibition zone using a zone scale [10]. All antimicrobial assays were conducted in triplicate (*n* = 3) and repeated in two independent experiments. Data are presented as the mean ± standard deviation (SD) of six measurements.

### 2.9. Antifungal Test Activity of Ag‐NPs

The fungus inhibition efficacy of Ag‐NP was assessed by diffusion assay against the aforementioned fungal strains that were enumerated by YPD medium and allowed to incubate for a duration of 3–5 days at 30°C [20]. A suspension of fungi was prepared in a sterilized phosphate buffer solution (PBS) with a pH of 7.0. The inoculum was adjusted to a concentration of 10^6^ spores/mL after counting in a cell counter chamber. Wells measuring 8 mm in diameter were created using a sterile cork borer. Subsequently, 60 *μ*L of Ag‐NP and AgNO_3_ was introduced separately into each well and allowed to incubate for 2 h at 4°C. Nystatin was used as the reference antifungal agent, and the plates were subsequently incubated for a time of 3 days at 30°C. Following reproduction, measurement and recording of inhibition zones were carried out [21].

### 2.10. Determination of Antioxidant Activity

The radical scavenging activity of Ag‐NP and vitamin C was assessed using the DPPH assay. Initially, 1 mL of Ag‐NPs at various concentrations (10, 20, 30, 40, 50, 60, and 100 *μ*g/mL) was mixed with 1 mL of 1 mM DPPH solution and vortexed. The mixture was then left undisturbed for 30 min at room temperature and in the dark. Subsequently, the absorbance was measured at a wavelength of 517 nm using the JENWAY 6800 spectrometer. DPPH served as a control, while methanol was used as the blank [10]. The percentage of inhibition of free radical scavenging activity was estimated using the formula: *%*of scavenging = [(*P*_c_ − *P*_s_)] × 100 where *P*_c_ is the absorbance of the control and *P*_s_ is the absorption of Ag‐NPs/vitamin C.

### 2.11. Cytotoxic Activity

After 24‐h growth period at 37°C with 5% CO_2_, the hepatocellular carcinoma cell line was introduced into flasks containing Dulbecco′s modified eagle medium (DMEM) enriched with 10% fetal bovine serum (FBS). After incubation, cells were trypsinized for 3–5 min at 800 rpm for 10 min to facilitate separation. For the experiment, a total of 96 wells were utilized in an ELISA plate, with 5000 cells in each well. The cells were allowed to grow into a monolayer, reaching a confluence of 70%–80% during 24‐h incubation at 37°C. The toxicity of Ag‐NP was tested at concentrations ranging from 10 to 50 *μ*g/mL in triplicate, and cell counting was performed under an optical microscope after 48 h to assess viability. A cytotoxicity solution was introduced into the culture medium to assess cell viability. Each well was treated with 200 *μ*L of MTT solution and then incubated for 4 to 5 h. Following the removal of the MTT solution, 200 *μ*L of DMSO was applied to each well in the absence of light for the subsequent incubation period. The optical density of the formazan product at 595 nm was determined using an ELISA reader from Bio‐Rad, United States [22].

### 2.12. Wound‐Healing Assay

The cell scratch assay was used to evaluate the wound‐healing potential of Ag‐NPs compared to the normal 3T3 fibroblast cell line. The experiment involved seeding 2 × 10^5^ cells/mL in a combination of M199 medium and DMEM with 10% phosphate buffer saline (PBs). Following cell seeding, the samples were placed in a CO_2_ incubator for 22–28 h. Once a monolayer confluence of approximately 70% to 80% was achieved, cells were scraped at the center of the culture well using sharp points. The damaged cells were then washed away with multiple fresh batches of media. Subsequently, the scratched wells were treated with the test samples (SNPs). The negative control was Hanks′ balanced salt solution (HBSS), while the positive control was allantoin, a commercial wound‐healing drug from Sigma‐Aldrich at a concentration of 50 *μ*g/mL. After removing the plates from the incubator, they were further incubated for 22–24 h to allow proper development. Phase‐contrast microscopy images documenting wound‐healing activity were captured after fixing and staining the cells [23].

### 2.13. Statistical Analysis

Statistical analysis was performed using IBM SPSS Statistics 23 for Windows, Version 23 (IBM Corp., Armonk, New York, United States). The experimental results were presented as means with standard errors (±SD). Following the one‐way ANOVA analysis, the significance set at *p* = 0.05 was reported.

## 3. Results

### 3.1. Fungal Isolation and Screening for Ag‐NP Synthesis

**Figure 1:**
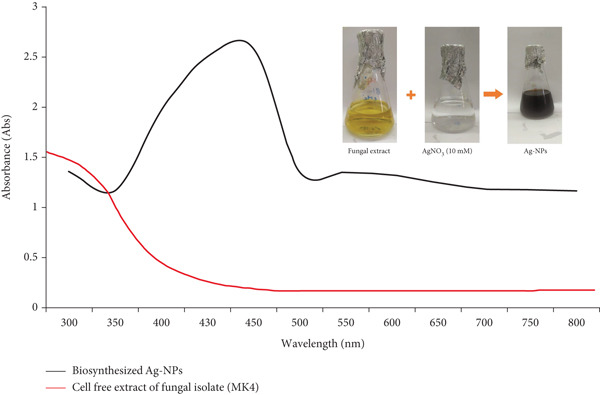
UV‐visible absorption spectrum of silver nanoparticles synthesized by the marine fungal isolate MK4. The surface plasmon resonance peak observed at 460 nm confirms the successful extracellular biosynthesis of Ag‐NPs.

The primary focus of the current study was the extracellular synthesis of Ag‐NPs using the supernatant of marine fungi. Of the 23 fungal isolates obtained from the sediment sample, their potential to generate Ag‐NPs was systematically evaluated. The screening process pinpointed a singular isolate, named MK4, which exhibited the ability to synthesize Ag‐NP. Interestingly, the reduction of Ag^+^ ions was visible after the addition of AgNO_3_ to the supernatant of marine fungi, leading to a distinct color change from yellow to dark brown. In the control group, no such alteration in color was observed (see Figure 1). This highlighted the specificity and effectiveness of the Ag‐NP synthesis process by the MK4 isolate.

### 3.2. Analysis by UV‐Visible Spectroscopy

A UV‐vis spectrophotometer was used to verify the existence of metal nanoparticles (see Figure 1). A wavelength peak at 460 nm was seen in the spectra of Ag‐NPs. The presence of this particular peak at 460 nm lends credence to the idea that Ag‐NPs were successfully synthesized in this study.

### 3.3. FTIR

**Figure 2:**
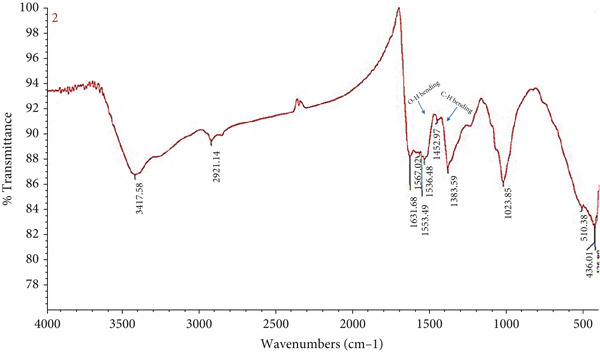
Fourier transform infrared spectra of silver nanoparticles biosynthesized by the marine fungal isolate MK4, indicating the presence of functional groups involved in nanoparticle reduction and stabilization.

The absorbance bands arranged between 400 and 4000 cm^−1^ were shown by the FTIR spectrum analysis. Figure 2 and Table 1 show that Ag‐NPs made from the marine fungus MK4 showed different bands. For Ag‐NP, the FTIR spectra revealed prominent absorption bands at 3417.58, 2921.14, 1631.68, 1553.49, 1452.97, 1383.59, and 1023.85 cm^−1^. A strong and wide absorbance peak at 3417.58 cm^−1^, which is the N‐H stretch, suggests the presence of amines. The strong medium absorbance peak at 2921.14 cm^−1^ (C‐H stretch) is associated with the alkane group. The band at 1631.68 cm^−1^ (N‐H bend) represents the characteristic feature of the amine group. The intense medium absorbance at 1553.49 cm^−1^ (N‐O stretching) is a characteristic of nitro compounds. The alkane group is assigned to the band at 1452.97 cm^−1^, which is a C‐H bending. The presence of the 1383.59 cm^−1^ band suggests that the (O‐H) vibrations are in a bending mode. As an added bonus, the stretching vibration of the carbon–oxygen bond is responsible for the bands observed at 1023.85 cm^−1^ in the FTIR spectra.

**Table 1:** FTIR absorption bands and their corresponding functional group assignments identified in silver nanoparticles biosynthesized by the marine fungal isolate MK4.

**Group No.**	**Group frequency cm** ^**−1**^ **of the sample**	**Functional group assignment**
1	3417.58	N‐H stretching (amine)
2	2921.14	C‐H stretching (alkane)
3	1631.68	N‐H bending (amine)
4	1553.49	N‐O stretching (nitro compounds)
5	1452.97	C‐H bending (alkane)
6	1383.59	O‐H bending (phenol)
7	1023.85	C‐O stretching (alkyl aryl ether)

### 3.4. XRD Analysis

**Figure 3:**
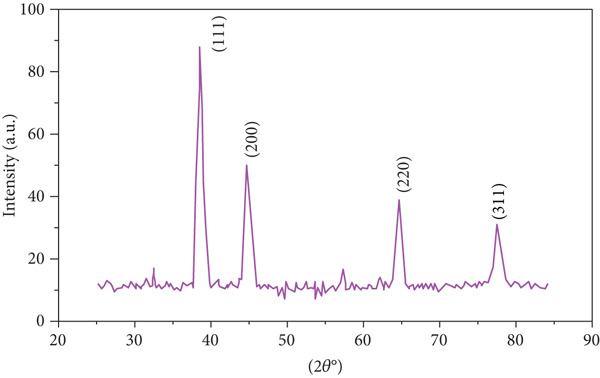
X‐ray diffraction pattern of silver nanoparticles biosynthesized by fungal isolate MK4, showing characteristic peaks corresponding to the face‐centered cubic (FCC) crystalline structure of metallic silver.

The crystallinity of the synthesized Ag‐NPs was investigated through the XRD patterns examination as shown in Figure 3. Four distinct peaks were observed at 2*θ* values recording 35.56°, 45.0°, 64.5°, and 77.64°. Bragg reflections of metallic (Ag‐NPs) formed in a face‐centered cubic (FCC) structure are completely matched by the different peaks seen in the XRD pattern. The Miller indices (hkl) were detected for each peak based on the crystallographic lattice planes. The indices were (111), (200), (220), and (311). These XRD spectra were matched with silver′s FCC structure (JCPDS File No. 04‐0783). The crystallite size of the synthesized Ag‐NPs was calculated according to Scherrer’s equation:

Crystallite size D=kλβCOSθ.

The crystallite size (*D*) is measured in nanometer, where *k* is Scherrer’s constant (0.9), *λ* is the wavelength of the x‐ray (0.15406 nm), and *β* is the full width at half maximum (FWHM) (radians) of the peak position (*θ*) (radians). The crystallite sizes of the Ag‐NPs were calculated in Table 2. The average crystallite size was around 20.7 nm, which is compatible with the TEM particle size.

**Table 2:** XRD peak positions (2*θ*), corresponding Miller indices (hkl), full width at half maximum (FWHM), and calculated crystallite sizes of silver nanoparticles biosynthesized by *Aspergillus flavus* MK4 using the Scherrer equation.

**Peak position (2** **θ** **)**	**hkl**	**FWHM** **β** **(°)**	**FWHM (** **β** **) (radians)**	**Cos** **θ**	**β**∗**C** **o** **s** **θ**	**Crystallite size** **D** **(nm)**
38.56	111	0.41	0.0072	0.94	0.0068	20.5
45.00	200	0.64	0.0112	0.92	0.0103	13.4
64.50	220	0.35	0.0061	0.85	0.0052	26.8
77.64	311	0.46	0.0080	0.78	0.0063	22.2

### 3.5. Analysis via TEM

**Figure 4:**
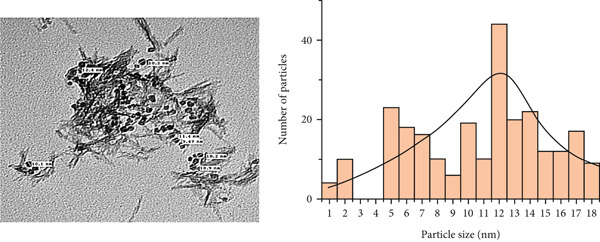
Transmission electron microscopy (TEM) image of silver nanoparticles biosynthesized by *Aspergillus flavus* MK4. The nanoparticles exhibit predominantly spherical morphology with an average diameter of 15 ± 0.8 nm. Samples were prepared by drop‐casting Ag‐NP suspension onto a carbon‐coated copper grid and imaged at 80 kV.

As shown in Figure 4, the TEM observation provided conclusive proof of the production of monocrystalline silver particles. The average particle size of Ag‐NP was 15 ± 0.8 nm, and its shape was mostly spherical, with uniform diameters. While a few instances of larger particles were identified in the sample, their overall quantity was considerably lower.

### 3.6. Sequencing the Most Effective Fungal Isolate (MK4)

**Figure 5:**
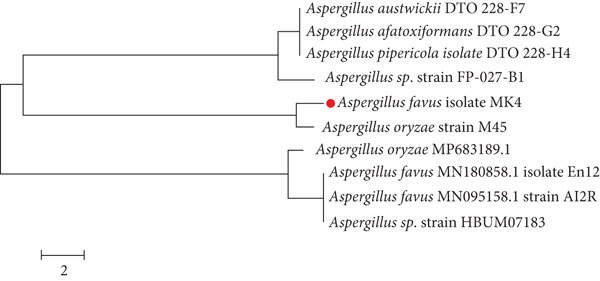
Phylogenetic tree of fungal isolate MK4 based on partial 18S rDNA sequence analysis, confirming its identification as *Aspergillus flavus*. The tree was constructed using (specify method, e.g., neighbor‐joining or maximum likelihood), with bootstrap values indicated at branch points.

During the detection phase, the most effective marine fungal isolate (MK4), which is capable of producing Ag‐NPs, was chosen. Phenotypic and molecular phylogenetic studies were used in the classification process. The fungal isolate MK4 was used to extract genomic DNA, which was then partially amplified from the 18S rRNA gene. The resulting amplicons were visualized through agarose gel electrophoresis. There was a perfect match between the 18S rRNA gene sequence and *A. flavus*. The *A. flavus* MK4 nucleotide sequence is stored in the GenBank database under the Accession Number OQ651270. Figure 5 illustrates the phylogenetic relationships between the experimental strains and their most closely related species.

### 3.7. Antibacterial Activity

Seven pathogenic bacteria, three of which were gram‐positive and four of which were gram‐negative, were tested for their antibacterial efficacy using the porous well diffusion technique in this study. Each well received 60 *μ*L of Ag nanoparticle solution, 1 mM silver nitrate, culture broth (without AgNo_3_) as a negative control, and ampicillin (1 mg/mL) as a positive control. The in vitro antibacterial activity of *A. flavus* MK4 is conducted and detailed in Table 3. The study showed that Ag‐NPs inhibited a wide range of gram‐positive and gram‐negative bacteria, demonstrating their broad‐spectrum antibacterial activity. The results suggest the presence of antibacterial compounds in the nanoparticles that could be explored for treating illnesses caused by these microorganisms. *S. aureus* ATCC 25923 and *P. fluorescens* DSM 50090 had the largest inhibitory zones (average diameter: 30 mm) among the strains examined, while *S. typhimurium* ATCC 14028 and *A. hydrophila* NRRL 914 had the smallest.

**Table 3:** Comparative antimicrobial activity of biosynthesized silver nanoparticles against selected gram‐positive and gram‐negative bacterial pathogens using the well diffusion assay. Results are expressed as mean inhibition zone diameters (mm ± SD) for Ag‐NPs, silver nitrate (AgNO_3_), culture filtrate (CF), and the positive control (ampicillin, 1 mg/mL).

**Pathogenic organism**	**CF**	**AgNO**_**3**_	**Ag-NPs**	**Ampicillin (1 mg/mL)**
*B. subtilis* ATCC 6633	0.0	10 ± 0.01^a^	28 ± 0.25^b^	44 ± 0.01^a^
*S. aureus* ATCC 25923	0.0	6 ± 0.21^b^	30 ± 0.2^a^	40 ± 0.01^a^
*B. cereus*	0.0	6 ± 0.01^a^	26 ± 0.01^b^	40 ± 0.01^a^
*P. aeruginosa* ATCC 9027	0.0	8 ± 0.5^a^	22 ± 0.2^b^	40 ± 0.01^a^
*S. typhimurium* ATCC 14028	0.0	6 ± 0.02^a^	14 ± 0.1^a^	42 ± 0.01^a^
*P. fluorescens* DSM 50090	0.0	12 ± 0.1^a^	30 ± 0.05^a^	40 ± 0.01^a^
*A. hydrophila* NRRL 914	0.0	4 ± 0.1^a^	14 ± 0.2^a^	40 ± 0.01^a^

*Note:* Means with different superscript letters in the same row are deemed significantly different (LSD test, *p* < 0.05), and triplicate measurements of two separate experiments were averaged with ±SD.

Abbreviations: Ag‐NPs, silver nanoparticles; AgNO_3_, silver nitrate; CF, culture filtrate.

### 3.8. Antifungal Activity

By employing the time‐tested agar well diffusion method, the research tested the antifungal effectiveness of biosynthesized Ag‐NPs against several fungus strains. Table 4 indicates that Ag‐NPs were highly effective against all fungi tested, suggesting that they could be useful as antifungal agents. Ag‐NPs had the ability to prevent fungal proliferation in *A. fumigatus*, *A. niger*, *A. flavus*, *A. terreus*, *A. parasiticus*, *P. oxalicum*, *F. solani*, *F. oxysporum*, and *C. albicans*, where the inhibition areas were 16 ± 0.12, 22 ± 0.1, 20 ± 0.02, 18 ± 0.01, 16 ± 0.02, 14 ± 0.04, 16 ± 0.1, 20 ± 0.1, and 18 ± 0.2 mm, respectively. The effect of AgNO_3_ on the fungi was limited, and in addition, no impact of nystatin was observed on any type of pathogenic fungi.

**Table 4:** Antifungal activity of biosynthesized silver nanoparticles against selected pathogenic fungal isolates as determined by the agar well diffusion method. Data represent mean inhibition zone diameters (mm ± SD) for Ag‐NPs, silver nitrate (AgNO_3_), and the positive control (nystatin).

**Pathogenic fungi**	**AgNO**_**3**_	**Ag-NPs**	**Nystatin**
*A. fumigatus* (NIOF‐F3)	8 ± 0.01^a^	16 ± 0.12^b^	0.0
*A. niger* (NIOF‐F8)	4 ± 0.21^b^	22 ± 0.1^a^	0.0
*A. flavus* (NIOF‐F12)	0.0	20 ± 0.02^b^	0.0
*A. terreus* (NIOF‐F13)	0.0	18 ± 0.01^b^	0.0
*A. parasiticus* (NIOF‐F15)	8 ± 0.02^a^	16 ± 0.02^b^	0.0
*P. oxalicum* (NIOF‐F22)	10 ± 0.1^a^	14 ± 0.04^a^	0.0
*F. solani* (NIOF‐F48)	4 ± 0.1^b^	16 ± 0.1^b^	0.0
*F. oxysporum* (NIOF‐F63)	6 ± 0.1^a^	20 ± 0.1^a^	0.0
*C. albicans* (NIOF‐F71)	10 ± 0.1^a^	18 ± 0.2^a^	0.0

*Note:* Means with different superscript letters in the same row are deemed significantly different (LSD test, *p* < 0.05), and triplicate measurements of two separate experiments were averaged with ±SD.

Abbreviations: Ag‐NPs, silver nanoparticles; AgNO_3_, silver nitrate.

### 3.9. Antioxidant Activity

The results indicated that both vitamin C and Ag‐NPs could function as antioxidants. Specifically, Ag‐NPs exhibited an antioxidant activity of 47.5%, surpassing the 29.4% antioxidant activity observed with vitamin C. These findings highlight the significantly superior antioxidant activity of Ag‐NPs compared to vitamin C.

### 3.10. Cytotoxic Activity Against Human Liver Cancer Cells (HepG‐2)

**Figure 6:**
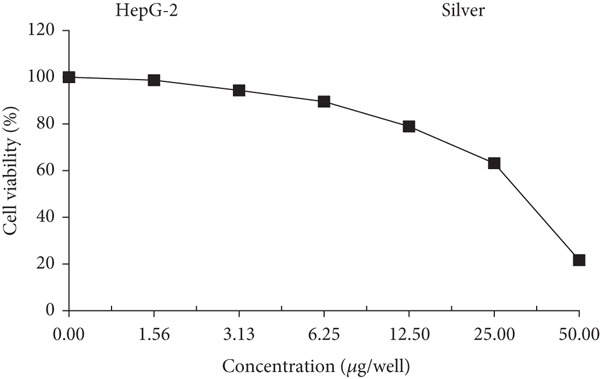
Dose–response curve showing the cytotoxic effect of silver nanoparticles biosynthesized by *Aspergillus flavus* MK4 on HepG‐2 cells after 24 h of exposure. The IC_50_ value was calculated based on cell viability assessed by the MTT assay.

Ag‐NPs synthesized by biological means were used in a cytotoxicity study against the HepG‐2 cell line to investigate their potential for the development of anticancer medications. The MTT assay was used to assess the percentage of viable HepG‐2 cells after exposure to biosynthesized Ag‐NPs at different concentrations. Figure 6 illustrates the varying percentages of viable cells at different concentrations of Ag‐NP. In particular, Ag‐NPs derived from *A. flavus* MK4 demonstrated a considerable level of anticancer activity, with IC_50_ values determined at 34.12 ± 1.05 (*μ*g/well) after 24‐h exposure.

### 3.11. Wound‐Healing Assay

**Figure 7:**
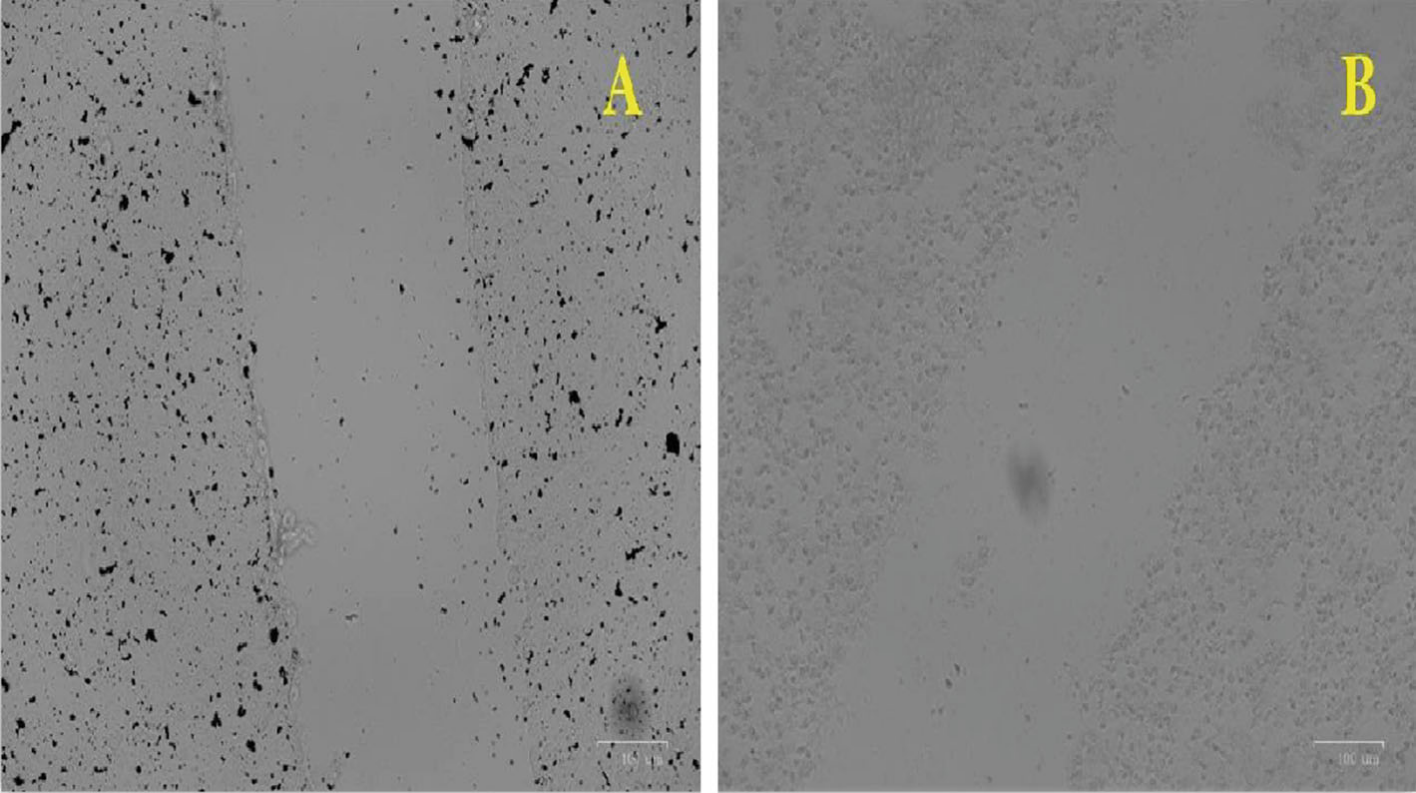
In vitro wound‐healing assay demonstrating the effect of silver nanoparticles synthesized by *Aspergillus flavus* MK4 on 3T3 fibroblast cells. (a) Untreated control cells showing baseline scratch area. (b) Cells treated with 25 *μ*g/mL Ag‐NPs after 72 h, showing enhanced migration and wound closure compared to control.

The investigation revealed that Ag‐NPs contributed to accelerated wound healing, as shown in Figure 7a,b. The proliferation of 3T3 fibroblast cells was monitored using the in vitro wound scratch method, capturing images of the scratched area at regular intervals to observe the impact of biogenic Ag‐NPs. The concentration of Ag‐NP was quantified using the MTT test, with a concentration of 25 *μ*g/mL Ag‐NP applied, which is below the IC_50_ value. Scar tissue healing occurred after Ag‐NP treatment due to the activation of fibroblasts and cell migration. Interestingly, after 72 h, the scar on the Ag‐NP‐treated plates had completely healed, reflecting the healing observed in the control group.

## 4. Discussion

Nanotechnology has recently become an interdisciplinary area, incorporating ideas and research from many other scientific and medical fields, such as mathematics, engineering, biology, materials science, physics, and chemistry [24]. A wide range of materials can be converted into nanoparticles by physical, chemical, and biological interactions. Biological methods for the creation of nanoparticles are the most encouraging of these technologies [25].

This study delves deeper into the realm of nanoparticle synthesis by focusing on the biological approach utilizing marine fungi specifically isolated from the Red Sea in Egypt. This method has significant advantages, eliminating the need for potentially harmful chemicals, extreme temperatures, or high pressures. This inherent simplicity leads to a straightforward and scalable process, paving the way for potential large‐scale production [26]. Their capacity to absorb and bioaccumulate heavy metals makes them ideal for reducing and stabilizing agents, and these organisms are often used for this purpose. Fungi, which are amenable to large‐scale cultivation, are also well suited to serve as “nanofactories,” facilitating the generation of nanoparticles with precisely manipulated size and shape [27]. Ag‐NP generation was achieved in this investigation by reducing aqueous Ag^+^ with the culture supernatants of marine fungal isolates at 28 ± 2^°^C. The brownish hue of the water‐based solution, caused by surface plasmon resonance (SPR) and reduction of AgNO_3_, served as an obvious indicator of the synthesis of Ag‐NPs [28]. As soon as the AgNO_3_ solution was added to the crude cell filtrate of the MK4, the color changed from yellow to dark brown. The culture supernatant devoid of AgNO_3_ did not undergo such an alteration, as shown in Figure 1. The clear change in color signified the obvious creation of Ag‐NPs. Even after 24 h of incubation, the color intensity of the AgNO_3_‐containing cell filtrate remained consistent, suggesting that the particles were evenly distributed in the solution [29].

Fungi have an advantage over other microorganisms due to their prolific production of proteins and enzymes. This attribute enables fungi to synthesize nanoparticles quickly and sustainably, making them a favorable choice for nanoparticle production [30]. Although several studies have focused on the biogenic manufacture of Ag‐NPs using fungus, our understanding of the underlying mechanisms is still limited. Enzymes found in fungal filtrate catalyze the extracellular creation of nanoparticles, which involves the conversion of Ag^+^ to Ag^0^ at the nanoscale. The emergence of plasmons on the colloidal surface indicates the creation of Ag‐NPs by isolating MK4. The alteration in color is a result of the reduction of Ag^+^ ions to metallic Ag facilitated by the reducing agents present in the fungal extract. Figure 1’s UV‐vis spectrum distinctly illustrates a singular localized surface plasmon resonance (LSPR) peak at 460 nm, confirming the successful synthesis of spherical Ag‐NPs, whereas there is no absorption band observed in the fungal extract solution. The clearly defined SPR absorption band and its narrow width indicate the narrow size distribution of the synthesized Ag‐NPs. Absorbance peaks at longer wavelengths imply the presence of larger nanoparticles, with these bands spanning from 400 to 460 nm [31]. A noticeable peak at 440 nm, which is the SPR band, was observed in the UV‐visible spectra of the cell filtrate produced by *A. terreus* with AgNO_3_. On the basis of these findings, it is highly probable that the solution contained Ag‐NPs [28].

Using FTIR spectroscopy to examine protein–Ag‐NP interactions demonstrated that biomolecules were pivotal in lowering Ag^+^ concentrations and stabilizing Ag‐NP amounts in the solution. The cell‐free extract, comprising biomolecules such as peptides, proteins, and carbohydrates, was analyzed within the range of 400–4000 cm^−1^ at a resolution of 4 cm^−1^. Figure 2 demonstrates how the stretching of primary amine (NH) was indicated by a clearly visible amine vibration band at 3417.58 cm^−1^. Furthermore, aromatic and aliphatic amine C‐N stretching vibrations were characterized by two bands at 1383.59 and 1023.85 cm^−1^, respectively. This work provided irrefutable evidence that proteins play a crucial role in maintaining the stability of the SNPs synthesized from the CFF of the selected isolate. The release of protein molecules outside the cell likely helped to enhance the synthesis and stability of Ag‐NPs [32]. The FTIR spectral examination of the microbial filtrate subjected to silver nitrate identified functional groups, which could have formed between amino acid residues and proteins produced during the creation of Ag‐NPs. Identifying peaks linked to particular functional groups, the research probably sheds light on how biomolecules interacted with Ag‐NPs throughout production [33]. Proteins play a crucial role in ensuring the stability of the generated nanoparticles; the FTIR spectra show peaks at 1629 and 1356 cm^−1^, confirming their presence. In proteins, the bands often represent the stretching of C‐C and C‐N bonds, respectively. This finding provides more evidence that proteins play a role in the process that stabilizes Ag‐NPs that are created [34]. Few new FTIR peaks emerged/intensified for the Ag‐NPs. For example, a band at approximately 510.38 and 436.01 cm^−1^ appeared for the Ag‐NP sample, which could be assigned to the bending vibrations of Ag‐O. These results are consistent with Ullah et al. [4] which showed in FTIR analysis of Ag‐NPs extracted from olive fruit biomass, which revealed a few peaks at 546 cm^−1^, suggesting they may be associated with bending vibrations at Ag‐O.

We used XRD and TEM to analyze the Ag‐NPs produced in depth, hoping to uncover new applications for them. In this study, the crystalline characteristics of Ag‐NPs were examined using XRD. Four diffraction peaks were seen at 2*θ* values of 35.56°, 45.0°, 64.5°, and 77.64°, respectively, representing the (111), (200), (220), and (311). These XRD spectra were matched with silver′s FCC structure (JCPDS File No. 04‐0783). The crystallite size of the synthesized Ag‐NPs was calculated according to Scherrer’s equation crystallite size (*D*) = *k* *λ*/*β*Cos*θ*, as shown in Figure 3. These peaks align with the standard diffraction pattern of the silver cubic structure, indicating a high‐quality crystal structure for the Ag‐NPs. Specifically, the presence of the planes (111), characterized by a peak at 28.8°, is typical for silver metal crystals. The cubic crystal structure, evident in the unit cell′s appearance, further confirms the quality of the Ag‐NPs. The XRD pattern of Ag‐NPs is characterized by several sharp peaks, which correspond to the different crystal planes of the silver lattice. The intensity of these peaks is related to the size and shape of the nanoparticles, as well as the degree of crystallinity. The position of the peaks can be used to determine the crystal structure of the nanoparticles. The XRD pattern of Ag‐NPs synthesized by *A. terreus* shows three main peaks at 38.1°, 44.3°, and 64.4°, which can be indexed to the (111), (200), and (220) reflections of the FCC crystal structure of silver, respectively. These peaks are consistent with the reported values for Ag‐NPs. The presence of these peaks confirms that the nanoparticles are crystalline and have a FCC structure. The FCC of these studies is consistent with previous studies on the x‐ray analysis of Ag‐NPs, which have shown similar patterns as Ejaz et al. The sharpness of the peaks indicates that the nanoparticles are relatively large and well crystallized. The absence of additional peaks suggests that the nanoparticles are pure silver and do not contain any other phases. The size of the nanoparticles can be estimated from the width of the diffraction peaks using the Scherrer equation. The Scherrer equation relates the width of a diffraction peak to the size of the crystallites that diffracted the x‐rays. In this case, the estimated size of the Ag‐NPs is approximately 20 nm. Overall, the XRD pattern is consistent with the formation of crystalline Ag‐NPs with a FCC structure and an average size of 20 nm.

To gain deeper insight into the structure and morphology of the Ag‐NPs, TEM analysis was conducted. Figure 4 shows electromagnetic transmission illustrations that show that the particles are of the same size and shape and are not clumped together. The particle sizes of Ag‐NP varied between 2 and 18 nm. According to San and Don′s [35] study on the green synthesis of Ag‐NP with the white rot fungus *Pycnoporus sanguineus*, the resulting particles are spherical and polydisperse and have a size range of 1 to 20 nm, as shown in TEM images. The average size of the silver particles produced by *A. terreus* ranged from 8 to 20 nm, according to a size distribution analysis that measured the diameters of 120 Ag‐NPs randomly selected in TEM images [36]. According to the investigation by Ammar and El‐Desouky, the dimensions of Ag‐NP ranged from 14 to 25 nm for *Penicillium expansum* and between 10 and 18 nm for *A. terreus* [37]. Based on the TEM result, the image shows several Ag‐NPs produced by *A. terreus*. The nanoparticles appear to be mostly spherical, with some variations in size and shape [38, 39]. The size of the nanoparticles ranges from about 10 to 20 nm in diameter. This is consistent with the size range reported in other studies of Ag‐NPs synthesized by *A. terreus*. The nanoparticles are mostly spherical, but some are slightly elongated or irregular in shape and relatively evenly distributed throughout the image with the same aggregation of nanoparticles. A slight discrepancy was observed between the particle size measured by TEM and that obtained via XRD. TEM analysis revealed an average particle size of 15 ± 0.8 nm, while XRD analysis yielded a crystallite size of approximately 20.7 nm based on the Scherrer equation. This variation can be attributed to the fundamental differences between the two characterization techniques. TEM provides a direct image of individual nanoparticles, revealing their physical size, while XRD measures the average size of coherently diffracting crystalline domains, which may include internal lattice defects or reflect polycrystalline structures. Therefore, XRD‐derived sizes often appear slightly larger than TEM values [4, 40]. Additionally, the presence of bio‐organic capping agents from the fungal extract may contribute to slight aggregation or the formation of thin surface coatings, which may affect the scattering pattern in XRD without significantly altering the visual size in TEM images [6]. Furthermore, sample preparation methods, including drying or electron beam exposure during imaging, may also influence the observed nanoparticle dimensions [35].

The comprehensive molecular investigations, coupled with the classical taxonomy outcomes, robustly affirmed the categorization of the isolated strain as *A. flavus*. You may find the nucleotide sequence in the genomic library with the Code OQ651270. Figure 5 illustrates the phylogenetic connections among the representative experimental strains and their closest species counterparts. The capacity of *Aspergillus* spp. to quickly produce stable particles makes them an attractive strain for the extracellular manufacture of metal nanoparticles, according to this research. *Aspergillus* spp. has been repeatedly named a strong candidate for the synthesis of Ag‐NPs in a plethora of investigations [41–46].

In general, Ag‐NPs exhibited effective inhibition against a range of bacterial species, although with varying degrees of efficacy. Contrary to the antibacterial impact being contingent on the gram classification (positive or negative), our research findings indicate that the effectiveness of Ag‐NPs is more closely tied to the specific bacterial species. This variability is likely attributed to the distinct membranes and cell walls inherent in different bacterial species. During the screening against various bacteria, the most potent effects were observed against *S. aureus* and *Proteus mirabilis*, with *B. subtilis* third. Ag‐NP antibacterial activities were more strongly resisted by *S. typhimurium* and *A. hydrophila* than by any of the other bacterial species. The size has a significant impact on the effectiveness of Ag‐NPs in suppressing bacteria [32, 35]. More evidence that the antibacterial effectiveness of nanoparticles depends on their particular forms has come from studies that looked at how different‐shaped nanoparticles inhibited bacterial growth. The influence of Ag‐NPs on bacterial cells is determined to be contingent on their shape, resulting in various outcomes depending on the morphologies of the nanoparticles. According to a number of studies, Ag‐NPs can impede cellular respiration by adhering to cell membrane surfaces, which can disrupt membrane permeability. The larger surface area of smaller Ag‐NPs makes them more effective at killing bacteria than bigger nanoparticles [47]. It is possible that Ag‐NPs could penetrate bacterial cells via interactions with their membrane. Because of their size, surface characteristics, and other unique qualities, Ag‐NPs can interact with bacterial membranes, changing their permeability and integrity. Nanoparticles may be able to enter the bacterial cell more easily if this interaction disrupts the membrane structure. When entering the cell, Ag‐NPs can exert their antibacterial properties in various ways, including disrupting cellular processes, producing oxidative stress, or interacting with vital biomolecules. The precise mechanisms of action can differ according to factors such as the size, shape, and surface chemistry of the nanoparticles, in addition to the distinct characteristics of the bacterial species in question [48]. The enormous surface area of Ag‐NPs, in comparison to other salts, is undoubtedly responsible for their strong antibacterial action. The increased surface area offers greater possibilities for interaction and contact with microbiological organisms. This improved contact enables a stronger influence on the internal structures and membranes of microbial cells, resulting in increased antibacterial effectiveness.

The exceptional antibacterial efficacy of Ag‐NPs can be attributed in large part to their unique physicochemical characteristics, such as their dimensions, morphology, and surface properties. Due to their ability to interact extensively with microbial surfaces and potentially infiltrate cells, they exhibit effectiveness in altering bacterial structures and functions. The significant ratio of surface area to volume is a crucial determinant that distinguishes Ag‐NPs from other salts in relation to their antibacterial characteristics [49]. The nanoparticles have the capacity to penetrate both the bacterium and the cell membrane. Ag‐NPs demonstrate a strong attraction to phosphorus‐containing molecules, such as DNA, as well as proteins that include sulfur, which are present in bacterial membranes. Upon entry of Ag‐NPs into the bacterium, a localized region of reduced molecular weight becomes evident within the cell. The bacteria gather here to shield the DNA from the ions of silver. Nanoparticles cause cell division and eventually cell death by targeting the respiratory chain. Because Ag^+^ are released intracellularly, nanoparticles have enhanced bactericidal activity [50]. Unlike gram‐positive bacteria, which possess a denser peptidoglycan layer impeding the entry of Ag‐NPs into their cells, research indicates that Ag‐NPs exhibit heightened toxicity toward gram‐negative bacteria. This increased toxicity is attributed to Ag‐NPs that easily penetrate the thinner outer membrane of gram‐negative bacteria and cause damage to their proteins and DNA. A higher concentration of Ag‐NPs is necessary to cause death in gram‐positive bacteria because their peptidoglycan covering is thicker [51]. The subsequent zones of inhibition were reported in the research conducted by Narasimha et al. on the antibacterial capabilities of Ag‐NPs generated from mushrooms: *Bacillus* sp., 1.9 cm; *Escherichia coli*; *P. aeruginosa*; and *S. aureus* all fall into this range. Particles with a larger contact surface area, such as those between 10 and 20 nm in size, are more efficient against both gram‐positive and gram‐negative bacteria, according to the study′s authors [52]. Similarly, at a concentration of 100 *μ*g mL^−1^, Ag‐NPs made from the water‐based extract of *A. flavus* showed significant antibacterial and antifungal effects against various microorganisms, including *S. aureus*, *B. subtilis*, *P. aeruginosa*, *E. coli*, *C. albicans*, *Candida glabrata*, *Candida tropicalis*, and *Candida parapsilosis* [53]. The findings of Fouda and his team indicate that the application of Ag‐NPs produced by *Penicillium italicum* resulted in clear areas of suppression for different microbes. Specifically, treatment with these Ag‐NPs resulted in clear zones measuring 17.6 ± 2.2 mm for *S. aureus*, 19.5 ± 0.5 mm for *E. coli*, 35.0 ± 0.5 mm for *C. albicans*, and 35.6 ± 0.6 mm for *C. tropicalis*, all observed at a concentration of 100 ppm [53]. Several mechanisms have been suggested to explain the antibacterial activity of Ag‐NPs. Upon entry into bacterial cells, Ag‐NPs release Ag^+^, which interact with essential intracellular targets. These ions can inactivate vital enzymes by binding to thiol groups, disrupting bacterial metabolism [54]. They also interact with bacterial DNA, altering its structure and impairing its function [55]. Additionally, Ag^+^ ions can disturb membrane integrity, promoting the leakage of potassium ions, and interfere with the respiratory chain, leading to an increase in reactive oxygen species (ROS), which contributes significantly to bacterial cell death [56]. Additional antibacterial properties of Ag‐NPs have been documented, particularly their ability to inhibit biofilm formation. Kalishwaralal et al. [57] demonstrated that Ag‐NPs significantly suppressed biofilm development by over 95% in *P. aeruginosa* and *Staphylococcus epidermidis*, two pathogens commonly associated with persistent infections. Beyond this, Ag‐NPs have also been implicated in triggering apoptosis‐like effects in bacteria, as reported by Lee et al. [58]. These effects include early markers such as phosphatidylserine exposure and later‐stage indicators like DNA fragmentation [59].

The results of the effective dispersion technique showed that Ag‐NP inhibited the growth of the tested strains of fungi, including *A. fumigatus* (NIOF‐F3), *A. niger* (NIOF‐F8), *A. flavus* (NIOF‐F12), *A. terreus* (NIOF‐F13), *A. parasiticus* (NIOF‐F15), *P. oxalicum* (NIOF‐F22), *F. solani* (NIOF‐F48), *F. oxysporum* (NIOF‐F63), and *C. albicans* (NIOF‐F71). The data presented in Table 2 show that *A. niger* exhibited a higher susceptibility to Ag‐NPs compared to other fungal isolates, displaying an inhibition zone of approximately 22 ± 0.1 mm with an optimal volume of 60 *μ*L of the Ag‐NP solution prepared. The antifungal properties of metallic nanoparticles have recently attracted attention [60, 61]. These nanoparticles include silver, zinc, copper, titanium, and others. Elbahnasawy et al. [54] noted the anticandidal activity of Ag‐NPs. Ag‐NP biosynthesized by endophytic *Rothia endophytica* against *C. albicans* was noted by Elbahnasawy et al. [62]. Ajaz et al. [63] tested ZV‐Ag‐NPs against the common plant disease *Colletotrichum falcatum* to see how effective they were as antifungals. In vitro comparisons between fungal mycelia and the control group revealed a significant inhibitory impact of ZV‐Ag‐NP at a dose of 20 *μ*g/mL. Regardless of the mycotoxigenic fungi *A. flavus* and *Aspergillus ochraceus*, Ag‐NP synthesized from cell‐free culture filtrates of *Penicillium chrysogenum* and *Fusarium chlamydosporum* showed significant antifungal efficacy [64]. At a dose of 100 *μ*g/mL, biogenic Ag‐NPs produced by the *Syzygium cumini* leaf extract show antifungal action against *A. flavus* and *A. parasiticus* strains of fungus [65].

The fungal cell wall comprises a rigid and resilient matrix accounting for approximately 40% of the cell’s volume, supported by an array of structural proteins and polysaccharides. Its outer surface is enriched with mannosylated glycoproteins containing modified N‐ and O‐linked oligosaccharides [66], while the inner layer predominantly consists of chitin and *β*‐(1→3)‐glucan, the latter comprising up to 60% of the cell wall’s dry weight. This inner layer is essential for withstanding internal turgor pressure and maintaining cellular integrity [67]. Ag‐NPs are believed to compromise this structural integrity by disrupting the cell wall, damaging surface proteins and nucleic acids, and generating ROS and free radicals. These actions may inhibit proton pumps and interfere with respiration by inducing ion efflux and impairing the electron transport chain [68]. The high surface‐area‐to‐volume ratio of smaller Ag‐NPs facilitates their penetration across fungal membranes, enhancing antifungal activity. Their toxicity is largely associated with ROS production, potentially leading to apoptotic‐like cell death. This cytotoxicity may arise from the synergistic or independent effects of Ag^+^ and Ag‐NPs themselves [69]. Nevertheless, the precise antifungal mechanisms of Ag‐NPs remain to be fully elucidated.

The antioxidant properties of Ag‐NPs are attributed to the adsorption of fungal components from a CFF onto the nanoparticles [70]. The Ag‐NPs exhibited an antioxidant activity of approximately 47.5%, surpassing the antioxidant activity of vitamin C, which was approximately 29.4%. The findings indicate that the antioxidant potential of Ag‐NPs exceeds that of vitamin C. Marine fungi, characterized by various biological traits, such as antioxidant, antibacterial, and antimalarial activities, are proficient in producing metal nanoparticles. Here, the seaweed‐isolated marine endophytic fungus *Cladosporium cladosporioides* has produced Ag‐NPs that are rich in antioxidants [19]. This discovery offers compelling evidence to support the effective use of Ag‐NPs as natural antioxidants, capable of providing protection against oxidative stressors and the degenerative diseases with which they are associated [71].

Biogenic Ag‐NPs demonstrate impacts on tumor cells that extend beyond their widely recognized antibacterial properties [29]. Figure 6 shows that the viability of a tumor cell line is reduced by Ag‐NPs in a dose‐dependent manner. More specifically, the HepG‐2 cell line was shown to be resistant to the anticancer effects of Ag‐NPs produced by *A. flavus* MK4. Researchers Husseiny and colleagues attempted to determine whether Ag‐NPs produced by *F. oxysporum* had any antibacterial or anticancer effects. The effectiveness of these nanoparticles against *E. coli* and *S. aureus* was further demonstrated by their ability to limit the growth of a tumor cell line. Nanoparticles showed great cytotoxicity and the possibility of efficient tumor control when exposed to MCF‐7 cells, as evidenced by a low IC_50_ value of 121.23 *μ*g/cm^3^ [72]. Ag‐NPs produced by fungi demonstrated robust anticancer activity against various tumor cell lines, exhibiting IC_50_ values that align with those reported in prior studies [41, 43, 73–76]. To address the limitation of assessing cytotoxicity solely on cancerous cells, we propose future investigations that incorporate dual screening models involving both malignant and nonmalignant human cell lines. This approach would allow for a more comprehensive evaluation of the selectivity and biosafety profile of biosynthesized Ag‐NPs. Furthermore, coculture systems that simulate the tumor microenvironment could provide more physiologically relevant insights into the differential cytotoxic responses.

The primary objective of antitumor therapy is to suppress the growth of cancer cells and promote their elimination, ideally without harming adjacent healthy tissues. Traditional treatment strategies utilize agents that interfere with cell cycle regulation and exert cytotoxic effects; however, these often result in severe adverse reactions and are frequently ineffective against resistant tumor types [77, 78]. In this context, Ag‐NPs have emerged as promising alternatives, owing to their distinctive physicochemical characteristics. Their ability to selectively target cancer cells enhances drug delivery and bioavailability, potentially improving therapeutic outcomes while reducing systemic toxicity [79]. The anticancer effects of Ag‐NPs, whether alone or in combination with chemotherapeutic agents, are mediated through several pathways including the induction of ROS, oxidative stress, DNA damage, cell cycle arrest, and the initiation of apoptosis and other forms of nonapoptotic cell death [80, 81].

In recent decades, Ag‐NPs have been widely investigated for their role in wound healing. Although Ag^0^ is generally inert and exhibits low cellular uptake in both mammalian and bacterial cells, it can be rapidly ionized in the presence of wound exudates. Once ionized, silver binds to proteins and cell membranes, where it becomes biologically active, effectively preventing biofilm formation and reducing the risk of wound infection [82]. In Figure 7a,b, we can see the results that demonstrate that Ag‐NPs accelerate the wound‐healing process. The potential of Ag‐NPs as a medicinal agent is enhanced by their demonstrated ability to repair wounds. The medicinal uses of Ag‐NPs in promoting skin regeneration and wound healing have been previously documented. Hence, the discovery of the promising wound‐healing potential of Ag‐NPs derived from a fungal extract holds great promise for biomedical applications. Fungus‐mediated biosynthesis of Ag‐NPs offers a biocompatible and eco‐friendly approach to enhancing wound healing. These mycogenic Ag‐NPs exert their therapeutic effect through multiple mechanisms. They exhibit potent antibacterial activity by disrupting microbial membranes, generating ROS, and preventing biofilm formation, thereby reducing infection risk, a major barrier to wound repair [83]. Additionally, Ag‐NPs modulate the inflammatory response by downregulating proinflammatory cytokines, facilitating transition to the proliferative phase [84]. They promote fibroblast and keratinocyte proliferation, enhance collagen deposition, and stimulate angiogenesis, all of which are critical for tissue regeneration [85]. Fungal metabolites involved in nanoparticle synthesis, such as enzymes and phenolic compounds, act as natural reducing and capping agents, which improve nanoparticle stability, biological activity, and antioxidant potential [40]. These combined effects position mycogenic Ag‐NPs as promising candidates in the development of advanced wound dressings.

## 5. Conclusions

X‐ray dispersion, UV‐vis spectroscopy, TEM, and FTIR were used to characterize the successfully synthesized Ag‐NPs that were made from the marine fungal strain *A. flavus* MK4. The nanoparticles that were created exhibited a SPR at 460 nm, and a TEM analysis revealed that there were Ag‐NPs measuring 15 nm in size. The study evaluated the antibacterial, antifungal, antioxidant, and cytotoxic properties of Ag‐NPs synthesized biologically. The results demonstrated the broad‐spectrum antibacterial activity of Ag‐NPs against both gram‐positive and gram‐negative bacteria, with notable inhibition zones observed particularly against *S. aureus* and *P. fluorescens*. Additionally, Ag‐NPs exhibited significant antifungal activity against various fungal strains, outperforming AgNO_3_ and nystatin. Moreover, Ag‐NPs demonstrated superior antioxidant activity compared to vitamin C. In terms of cytotoxicity, Ag‐NPs derived from *A. flavus* MK4 showed promising anticancer activity against HepG‐2 cells, indicating their potential in cancer therapy. Furthermore, Ag‐NPs accelerated wound healing by promoting fibroblast activation and cell migration. These findings suggest the potential of biogenic Ag‐NPs in various biomedical applications, including antimicrobial agents, antioxidants, and wound‐healing therapies.

**Nomenclature**
Ag‐NPs: silver nanoparticles
nm: nanometer
TEM: transmission electron microscope
HepG‐2: hepatoblastoma cell line Generation 2
MTT: 3‐[4,5‐dimethylthiazol‐2‐yl]‐2,5‐diphenyl tetrazolium bromide
DPPH: 2,2‐diphenyl‐1‐picrylhydrazyl
Ag^+^
: silver ions
UV‐vis spectroscopy: ultraviolet‐visible spectroscopy
XRD: x‐ray diffraction
FTIR: Fourier transform infrared spectroscopy
CFF: cell‐free filtrate
ATCC: American Type Culture Collection
DSM: Deutsche Sammlung von Mikroorganismen
NRRL: Northern Regional Research Laboratory
NIOF: National Institute of Oceanography and Fisheries
YPD: yeast extract peptone dextrose
°C: degree Celsius
%: percentage
MPDA: modified potato dextrose agar
g: gram
rpm: rotation per minute
ml: mill
mmole: mill mole
DNA: deoxyribonucleic acid
NCBI: National Center for Biotechnology Information
*μ*l: microliter
mg/mL: milligrams per milliliter
PBS: phosphate buffer solution
DMEM: Dulbecco’s modified eagle medium
FBS: fetal bovine serum
ELISA: enzyme‐linked immunosorbent assay
SE: standard deviation
*p*
: *p* value
FCC: face‐centered cubic
hkl: Miller indices
FWHM: full width at half maximum
IC_50_
: half‐maximal inhibitory concentration
SPR: surface plasmon resonance
LSPR: localized surface plasmon resonance

## Ethics Statement

The authors have nothing to report.

## Consent

The authors have nothing to report.

## Disclosure

This manuscript has been submitted as a preprint in the link https://www.preprints.org/manuscript/202312.1737/v1 [86].

## Conflicts of Interest

The authors declare no conflicts of interest.

## Funding

The authors would like to thank Ongoing Research Funding Program (ORF‐2025‐024‐1), King Saud University, Riyadh, Saudi Arabia, for the financial support.

## Data Availability

All the data are available in the manuscript.
